# Supplementary material for: Ageing affects DNA methylation drift and transcriptional cell-to-cell variability in mouse muscle stem cells
Source: Nat Commun. 2019 Sep 25;10:4361. doi: 10.1038/s41467-019-12293-4 (PMC6761124; doi:10.1038/s41467-019-12293-4)
Supplement: Supplementary file 1 — Supplementary Information [file 41467_2019_12293_MOESM1_ESM.pdf]

Ageing affects DNA methylation drift and transcriptional cell-to-cell variability in mouse muscle stem cells

Hernando-Herraez, Evano, Stubbs *et al.*, 2019

Supplementary Information

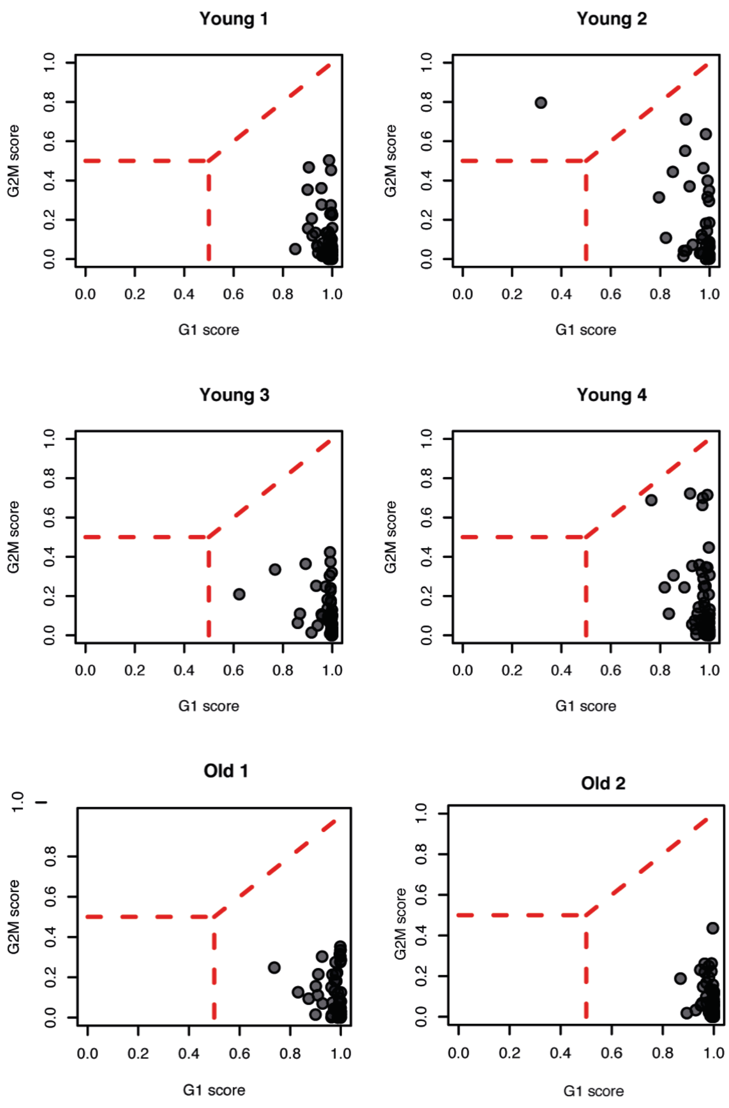

Supplementary Figure 1. Cell cycle stage of Pax7^{Hi} cells.

Scatter plots of predicted phases of the cell cycle (G1 score and G2M score) for single cells (grey circles) from young and old mice.

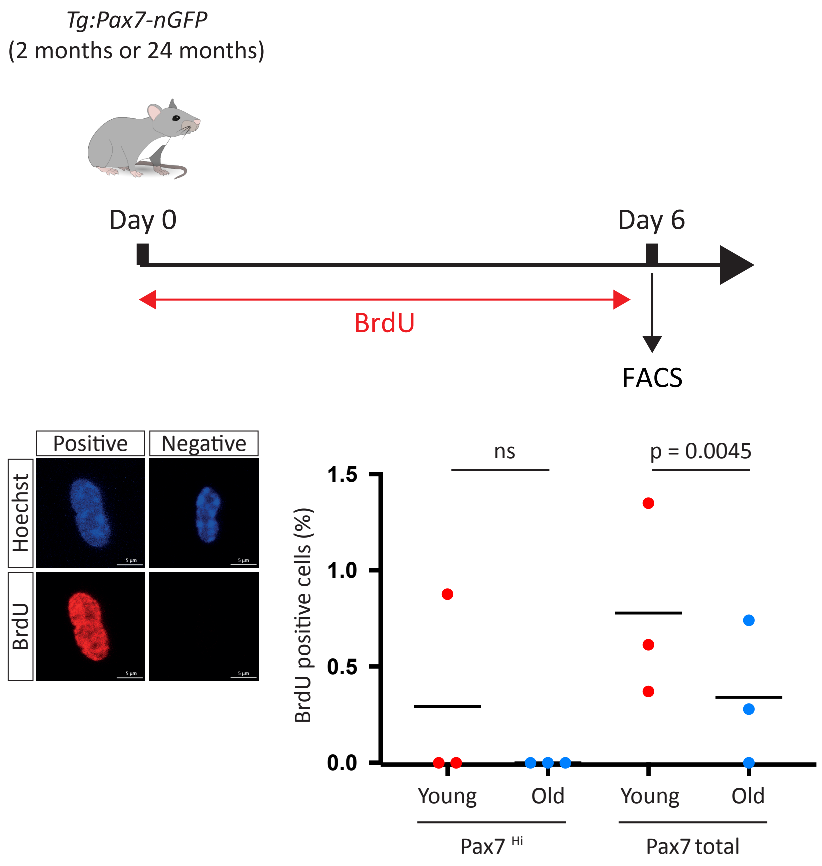

Supplementary Figure 2. *In vivo* proliferation of total and Pax7^{Hi} muscle stem cells in young and old mice.

BrdU was administered to young and old mice for 6 days to label cell cycle entry, and BrdU uptake was measured on isolated muscle stem cells (Pax7-nGFP^{Hi} subpopulation or Pax7-nGFP total population). BrdU uptake was detected at low frequency in Pax7^{Hi} cells (1 BrdU+ cell of 289 counted cells in young, 0 BrdU+ of 301 counted cells in old) with no significant difference between ages (Fisher exact test). BrdU uptake was higher (p=0.0045, Fisher exact test) in young total muscle stem cells (20 BrdU+ cells of 2886 counted cells) compared to old total muscle stem cells (5 BrdU+ cells of 2709 counted cells). Representative examples of BrdU+ and BrdU- are illustrated in images. Scale bar: 5 microns; n=3 old and n=3 young mice. The dotplot indicates individual values and the mean. Source data are provided as a Source Data file.

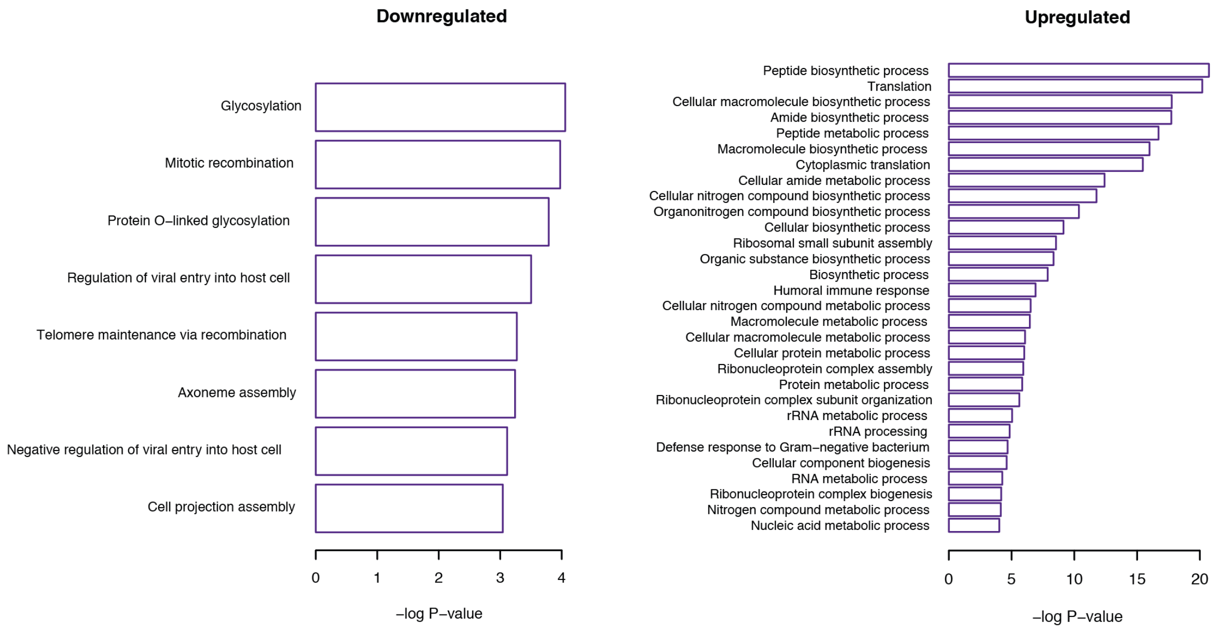

Supplementary Figure 3. P-values of the GO terms associated with genes that are differentially expressed with age, down regulated (left) and upregulated (right).

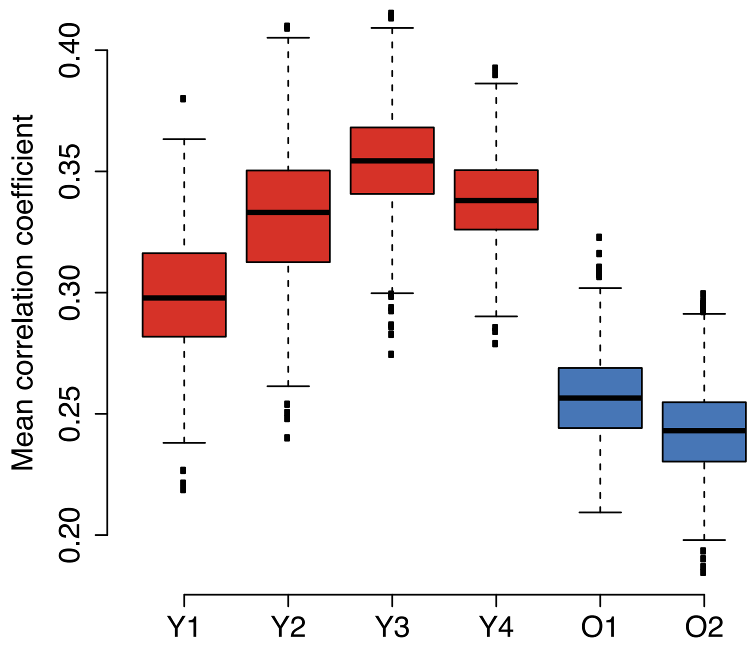

Supplementary Figure 4. Cell-to-cell transcriptional variability.

Distribution of the mean Spearman's correlation coefficient across cohorts of 10 cells over 1,000 iterations (P< 0.001). For all boxplots, the box represents the interquartile range and the horizontal line in the box represents the median (n =1,000).

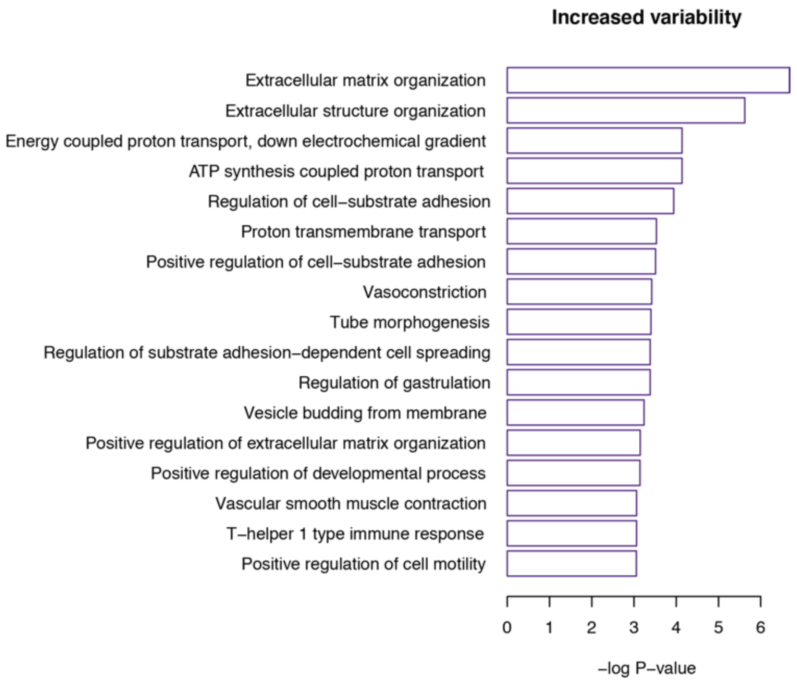

Supplementary Figure 5. P-values of the GO terms associated with genes with decreased expression frequency with age (expression frequency > 15%).

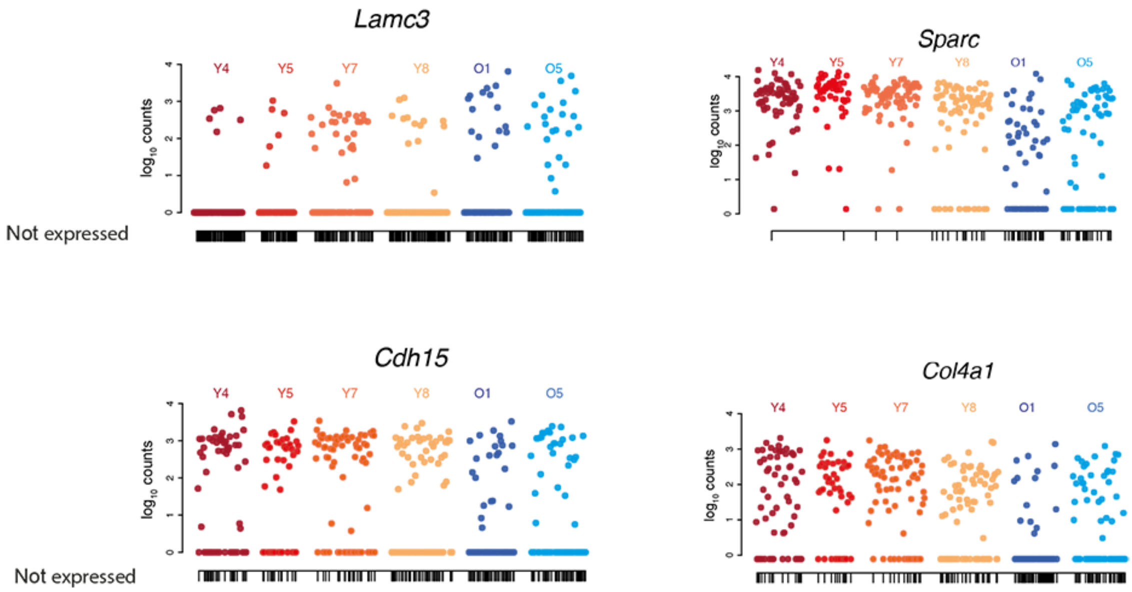

Supplementary Figure 6. Examples of transcriptional variability of selected extracellular matrix-related genes

Expression level of specific genes (y-axis) across different samples (x-axis). Each dot represents a cell. Vertical lines on the x-axis indicate cells that do not express the gene.

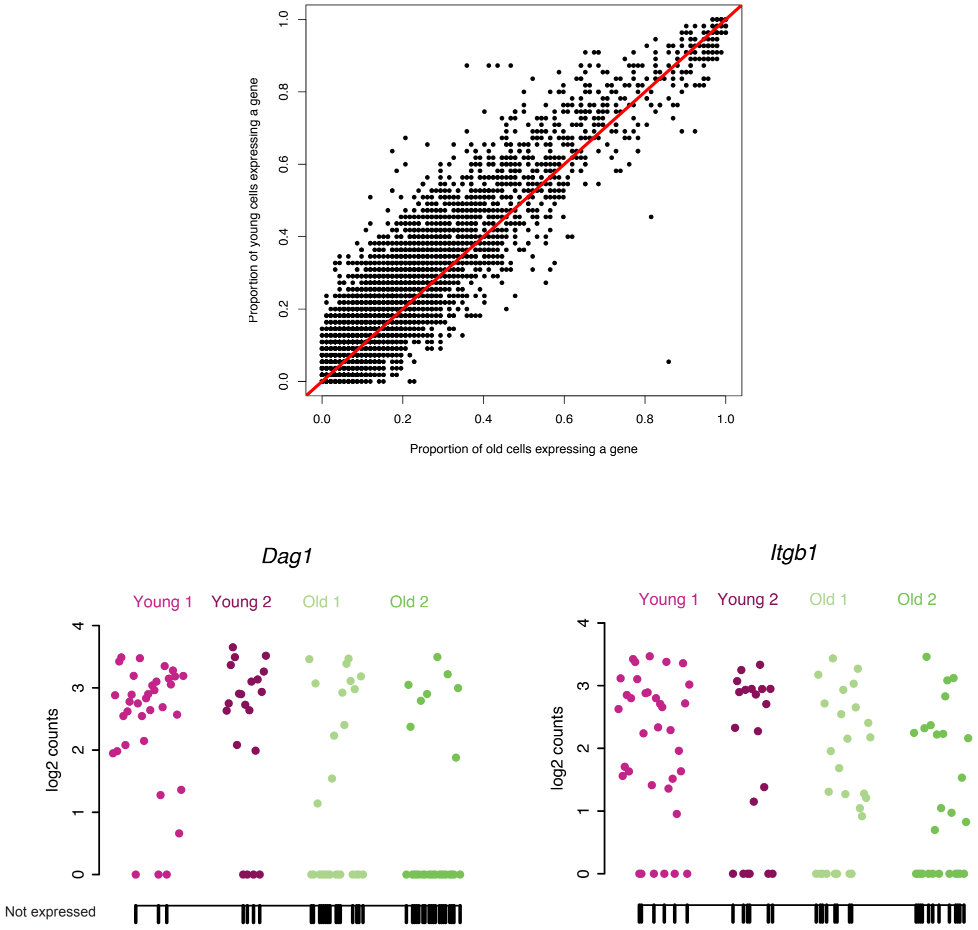

Supplementary Figure 7. Frequency of gene expression in young and old cells from the total Pax7-nGFP population measured by scRNA-seq (top). Expression levels of specific genes (y-axis) across different samples (x-axis) from the total Pax7-nGFP population. Each dot represents a cell. Vertical lines on the x-axis indicate cells that do not express the gene. Expression frequencies: *Dag1*: young 0.87, old 0.41; *Itgb1*: young 0.76, old 0.60.

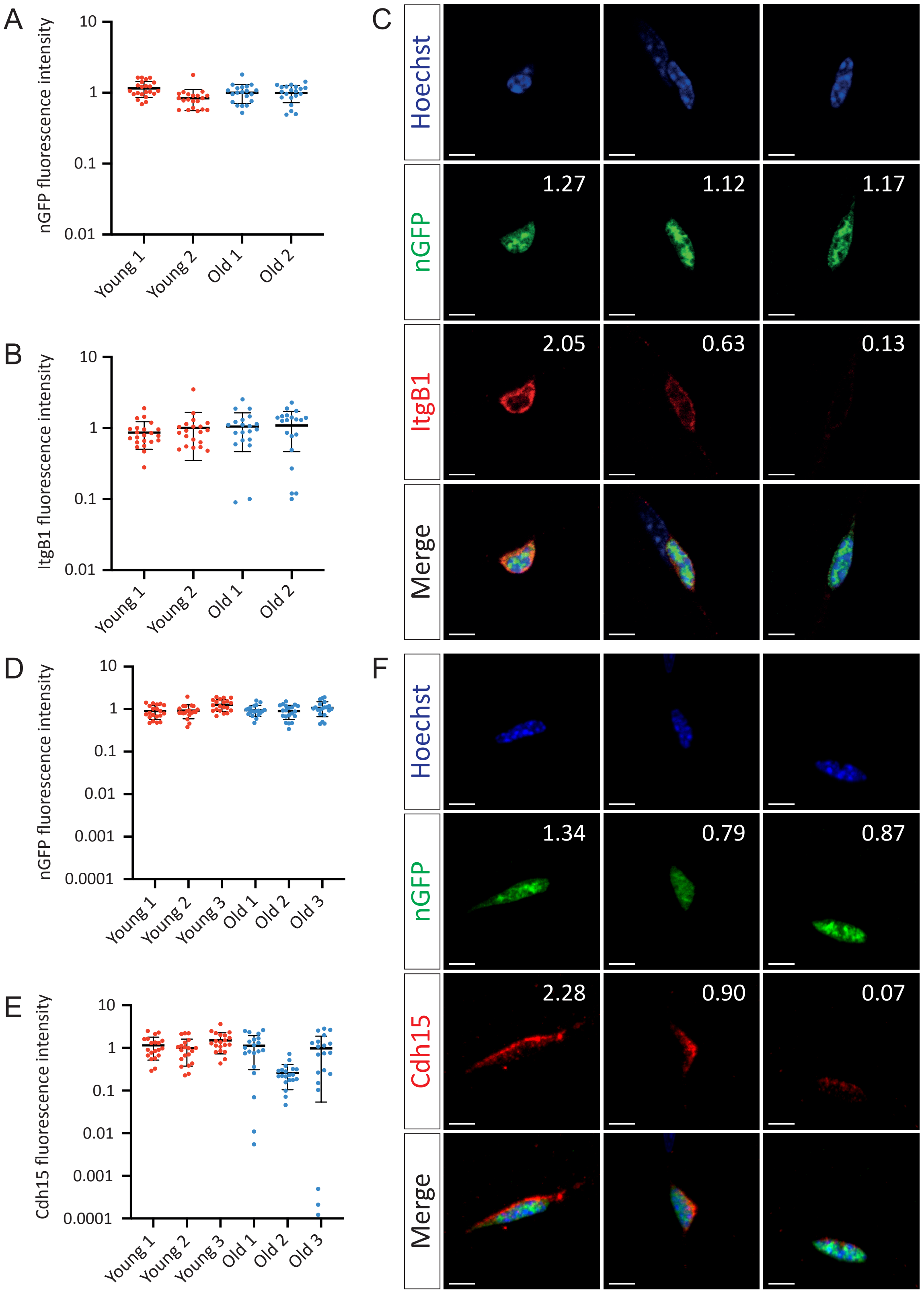

Supplementary Figure 8. Itgb1 and Cdh15 protein levels in young and old muscle stem cells.

The levels of nGFP, Itgb1 and Cdh15 proteins in quiescent muscle stem cells from young and

old *Tg:Pax7-nGFP* mice were measured after isolation of single myofibres and quantification of immunofluorescence intensity.

(A-C) Normalized total protein level of nGFP (A) and Itgb1 (B) from 2 young and 2 old individuals. 20 muscle stem cells were scored for each mouse. (C) Representative examples of muscle stem cells with different levels of nGFP and Itgb1 expression, with individual normalized values of total immunofluorescence intensity. Scale bar: 5 microns.

(D-F) Normalized total protein level of nGFP (D) and Cdh15 (E) from 3 young and 3 old individuals. 20 muscle stem cells were scored for each individual. Note that one of the three old individuals showed a global reduction in Cdh15 immunofluorescence intensity, which could reflect a global down-regulation of Cdh15 expression or a technical issue with this animal. (F) Representative examples of muscle stem cells with different levels of nGFP and Cdh15 expression, with individual normalized values of total immunofluorescence intensity. Scale bar: 5 microns. Dotplots indicate mean with standard deviation.

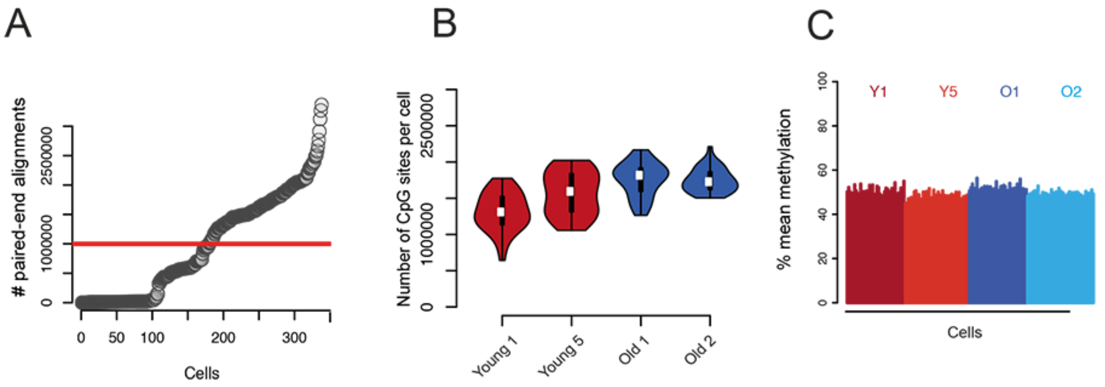

Supplementary Figure 9. Quality control of single-cell DNA methylation data.

(A) Number of pair-end alignments per cell. Cells below the threshold were excluded from the study.

(B) Number of CpG sites per cell and individual. Circles inside the violin plots represent the median of the data and the boxes indicate the interquartile range.

(C) Mean methylation per cell showing no global differences between ages.

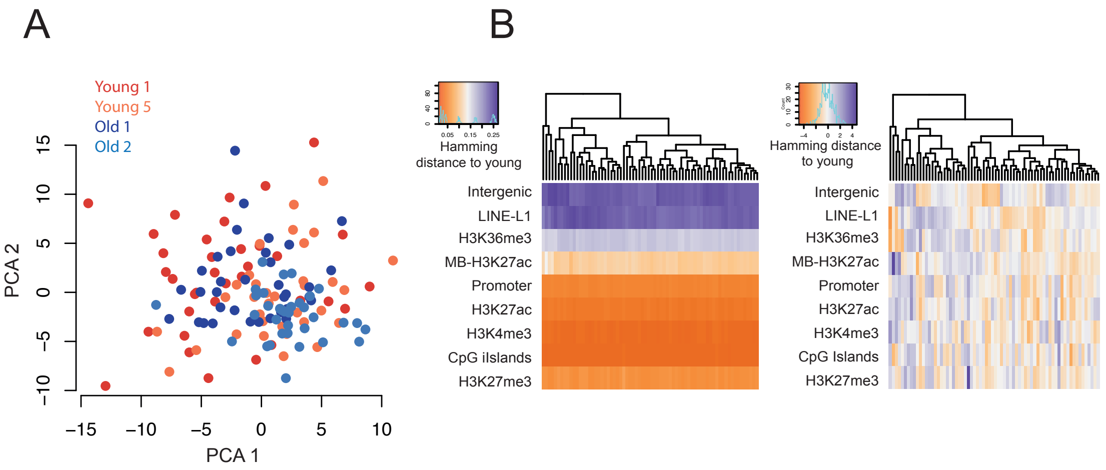

Supplementary Figure 10. Cell clustering based on DNA methylation data

(A) PCA on gene body methylation showing no clear differences between ages.

(B) Heatmap showing Hamming distances between the average methylation from young cells and individual old cells (columns) across different genomic context (rows) (left). Same measure normalised by genomic context (right) showing no cellular substructure.

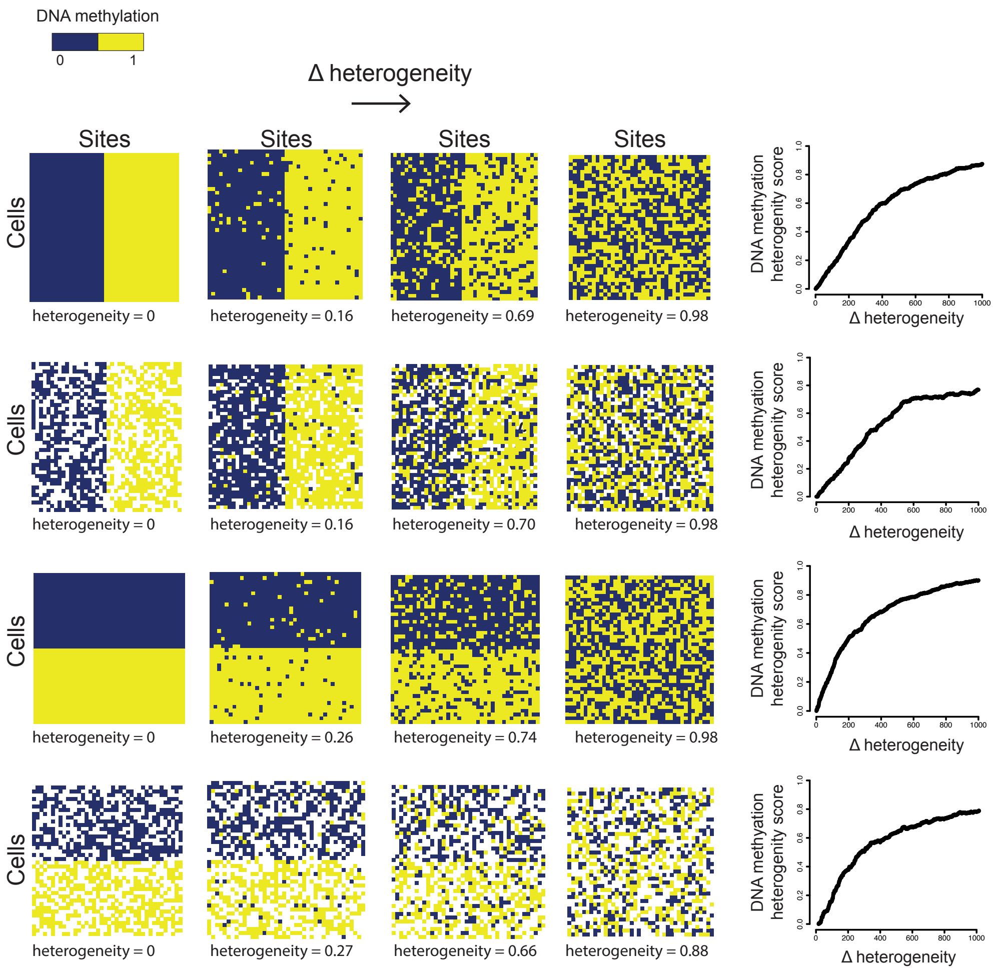

Supplementary Figure 11. DNA methylation heterogeneity on simulated data.

DNA methylation heterogeneity score tested with simulated data of increasing heterogeneity in four different scenarios. All scenarios have similar levels of DNA methylation but different population substructures and missing values: homogenous population (first and second rows) and clear subpopulations (third and fourth rows). Missing values are represented in white.

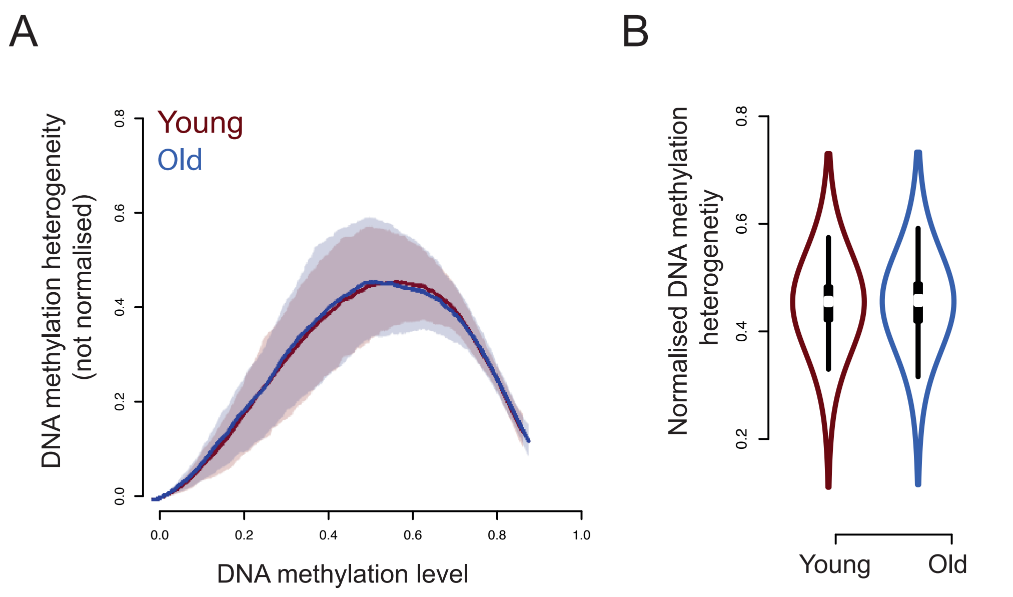

Supplementary Figure 12. Global levels of DNA methylation heterogeneity between ages.

(A) DNA methylation levels and methylation heterogeneity in young and old cells.

(B) Normalised DNA methylation heterogeneity in young and old cells. Circles inside the violin plots represent the median of the data and the boxes indicate the interquartile range.

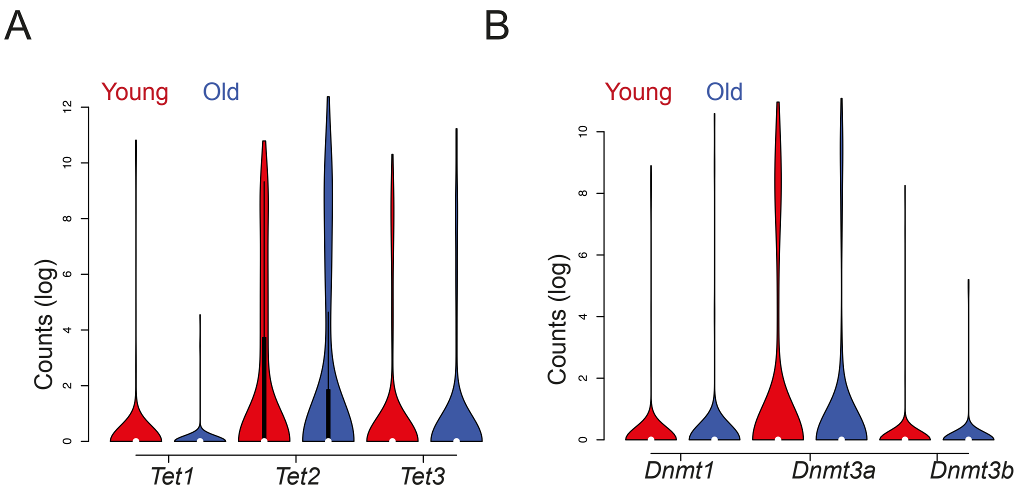

Supplementary Figure 13. DNA methylation enzymes show similar expression profiles.

(A) Expression levels of the enzymes for active demethylation in young and old samples.

(B) Expression levels of the DNA methylation enzymes in young and old samples. Circles inside the violin plots represent the median of the data and the boxes indicate the interquartile range.

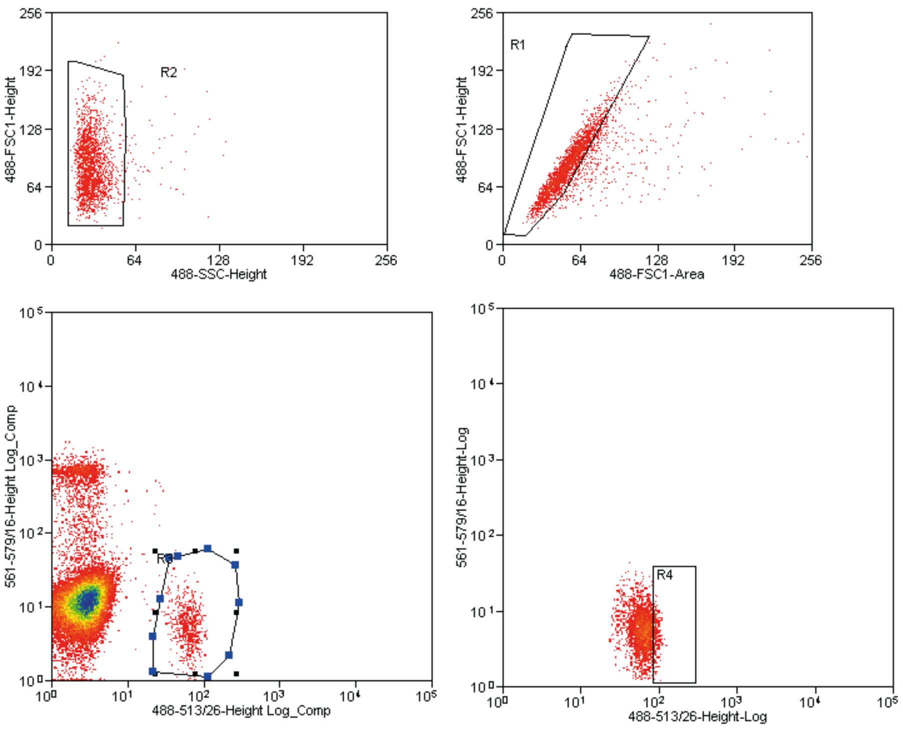

Supplementary Figure 14. Isolation of single muscle stem cells by FACS.

Muscle stem cells were isolated by FACS by gating first on size and granulosity (R2 gate), excluding doublets (R1 gate) and gating on the GFP^{+}/PI^{-} population (R3 gate). Total Pax7-nGFP cells (R3 gate) or Pax7-$nGFP^{Hi}$ cells (top 10% highest nGFP-expressing cells, R4 gate) were sorted as single cells.

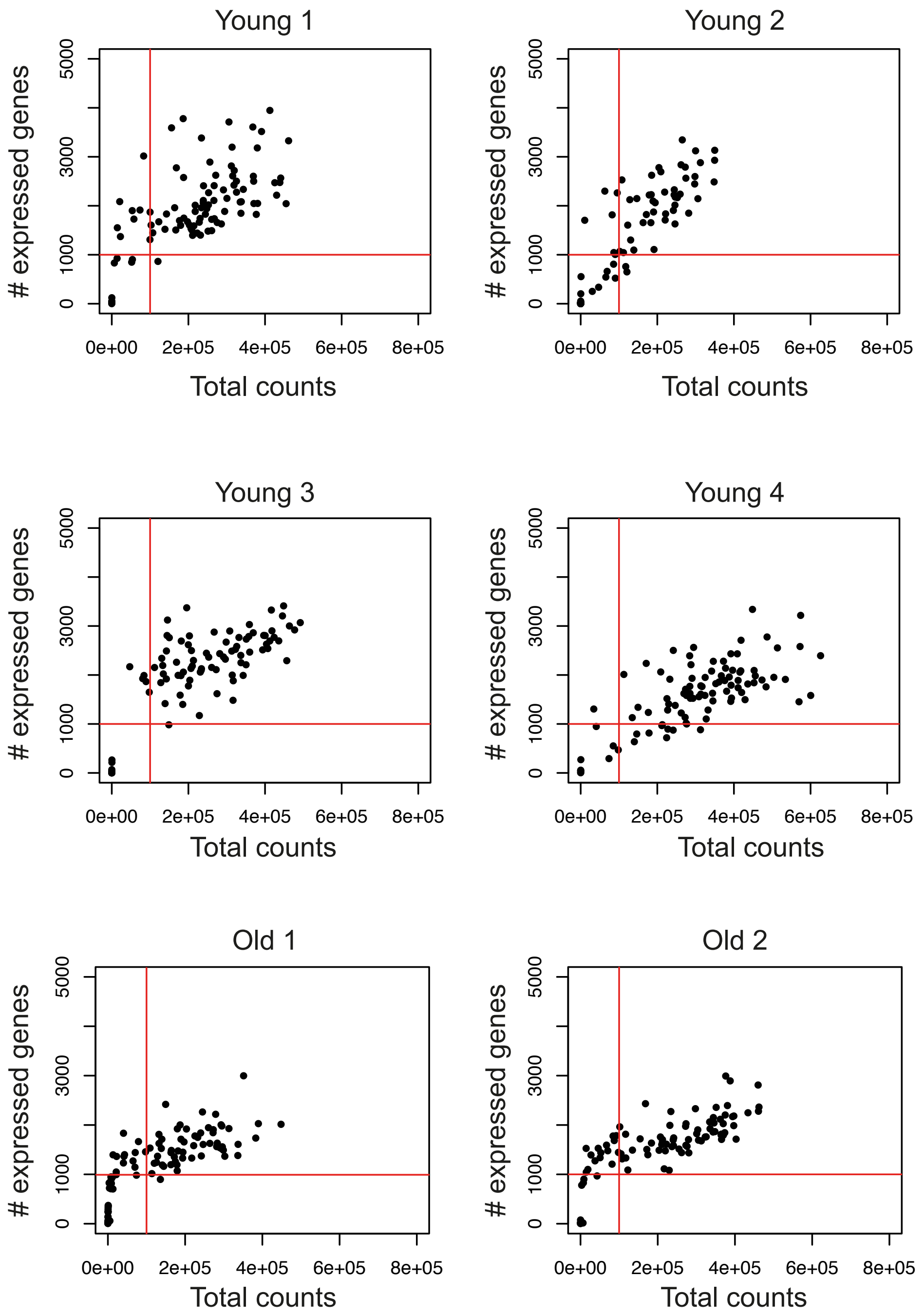

Supplementary Figure 15. Quality control of single-cell RNA-seq data.

Plot representing number of genes and total expression counts expressed in each cell per individual. Cells above highlighted threshold (1000 genes, 10^5 counts) were included in the study.

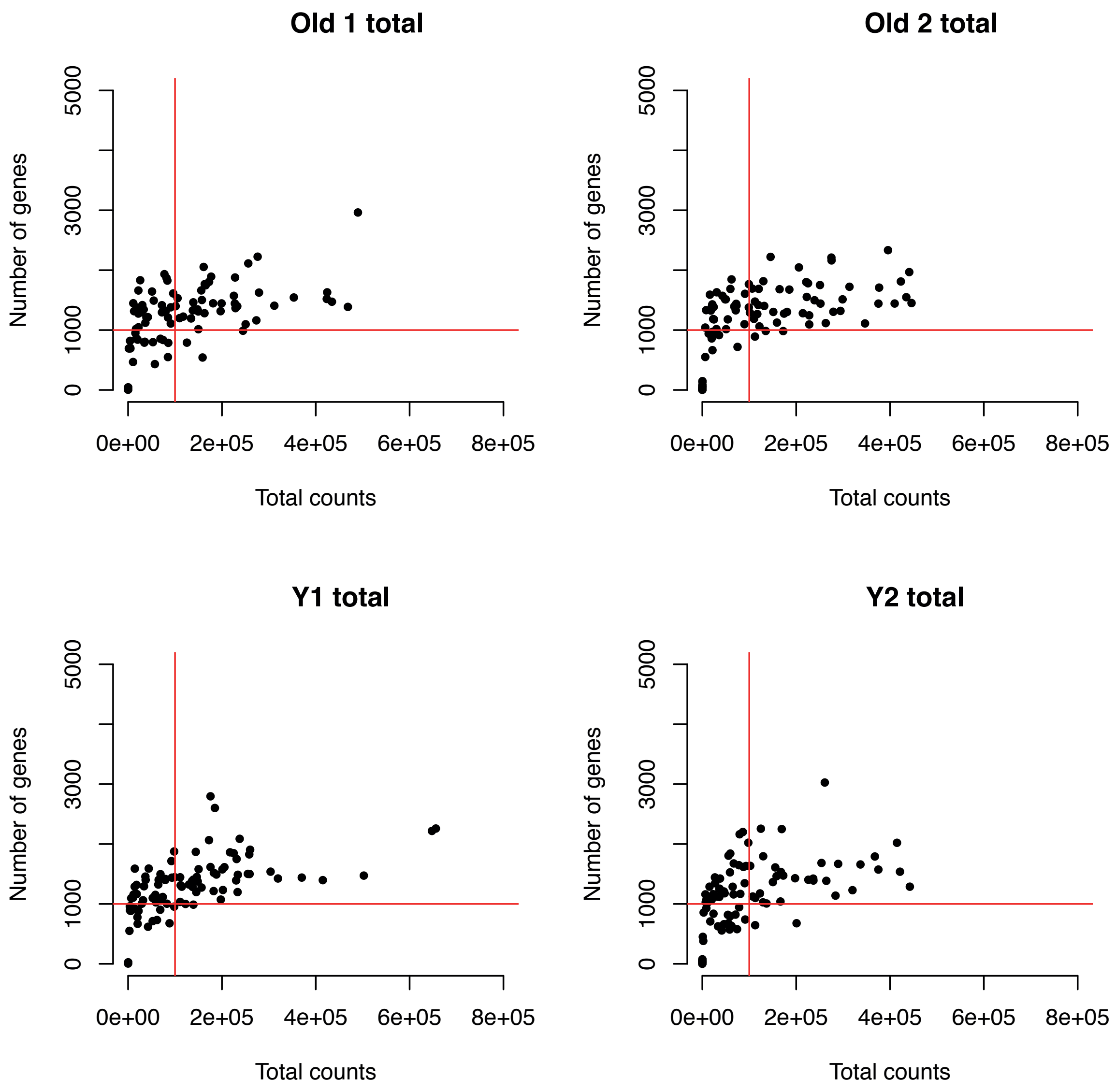

Supplementary Figure 16. Quality control of single-cell RNA-seq data from total Pax7-nGFP cell population.

Plot representing number of genes and total expression counts expressed in each cell per individual. Cells above highlighted threshold (1000 genes, 10^5 counts) were included in the study.

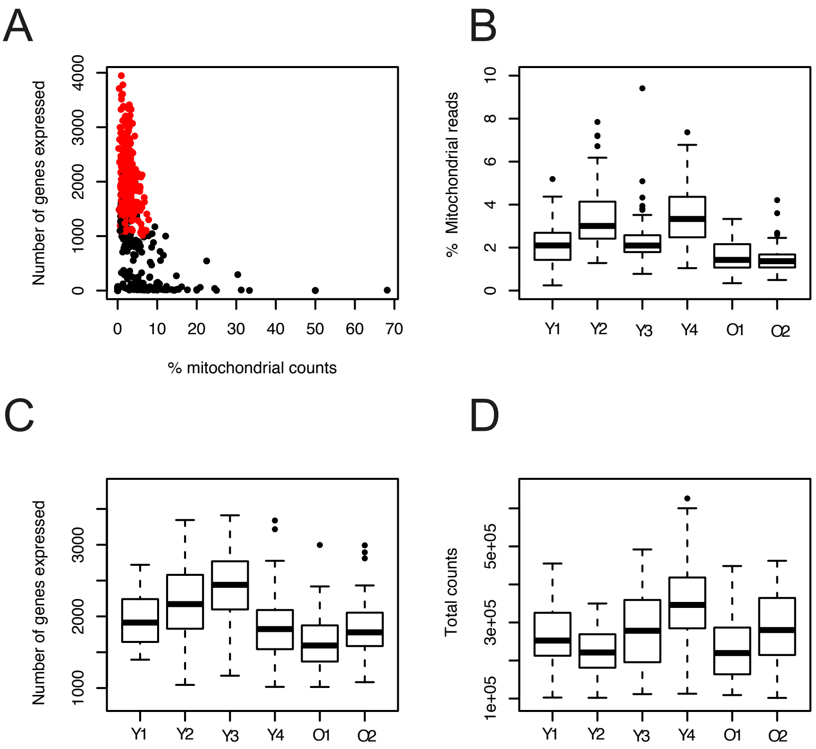

Supplementary Figure 17. Quality control of single-cell RNA-seq data.

(A) Plot representing the number of genes expressed per cell (y-axis) and the % of reads on mitochondrial reads (x-axis). Red dots represent the cells that passed the QC and were included in our study.

(B) Distribution of the reads assigned to mitochondrial genes per cell for each individual. For all boxplots, the box represents the interquartile range and the horizontal line in the box represents the median.

(C) Distribution of the number of genes expressed per cell for each sample. For all boxplots, the box represents the interquartile range and the horizontal line in the box represents the median.

(D) Distributions of total counts per cell for each sample. For all boxplots, the box represents the interquartile range and the horizontal line in the box represents the median.

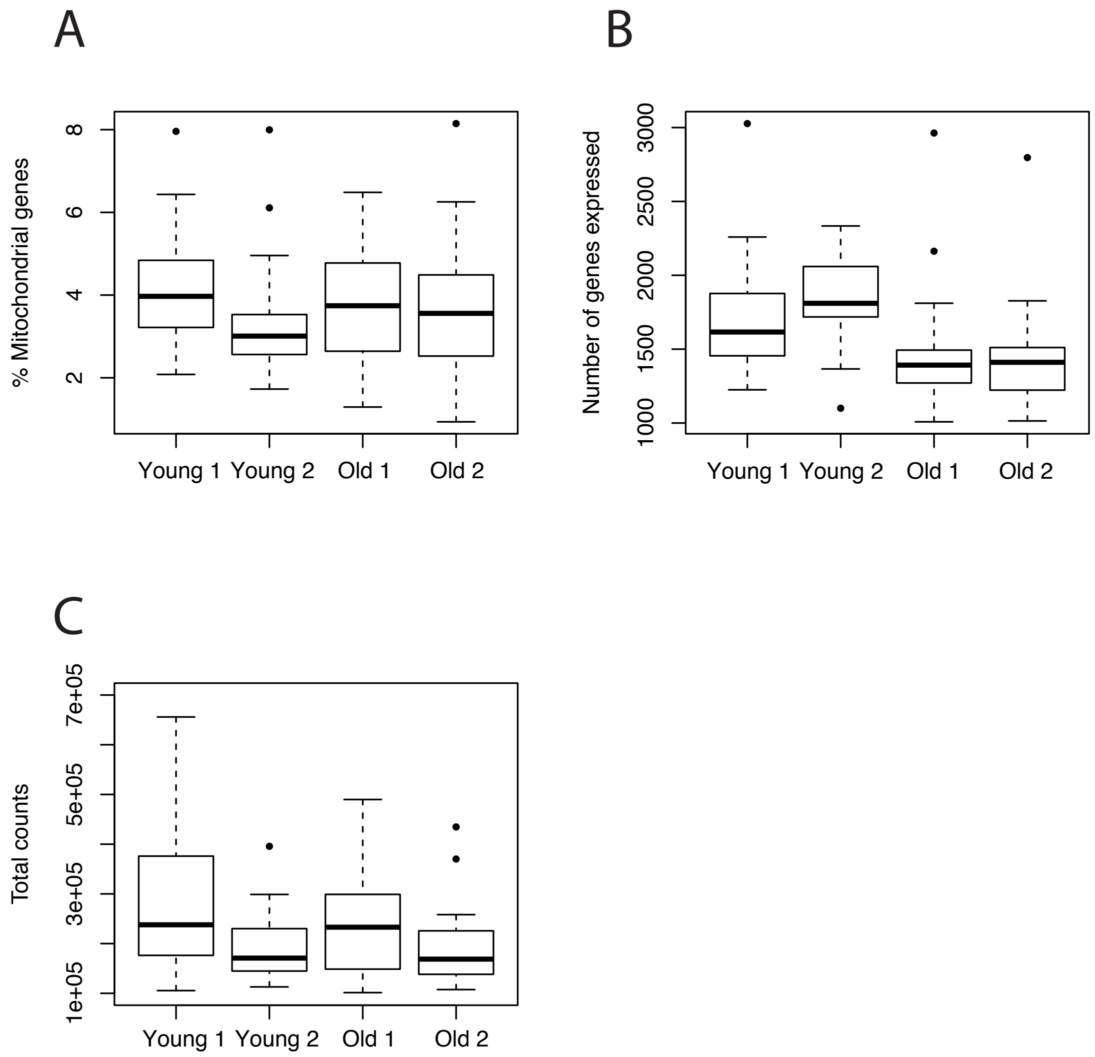

Supplementary Figure 18. Quality control of single-cell RNA-seq data of total Pax7-nGFP cell population

(A) Distribution of the reads assigned to mitochondrial genes per cell for each individual. For all boxplots, the box represents the interquartile range and the horizontal line in the box represents the median.

(B) Distribution of the number of genes expressed per cell. For all boxplots, the box represents the interquartile range and the horizontal line in the box represents the median.

(C) Distributions of total counts per cell for each sample. For all boxplots, the box represents the interquartile range and the horizontal line in the box represents the median.

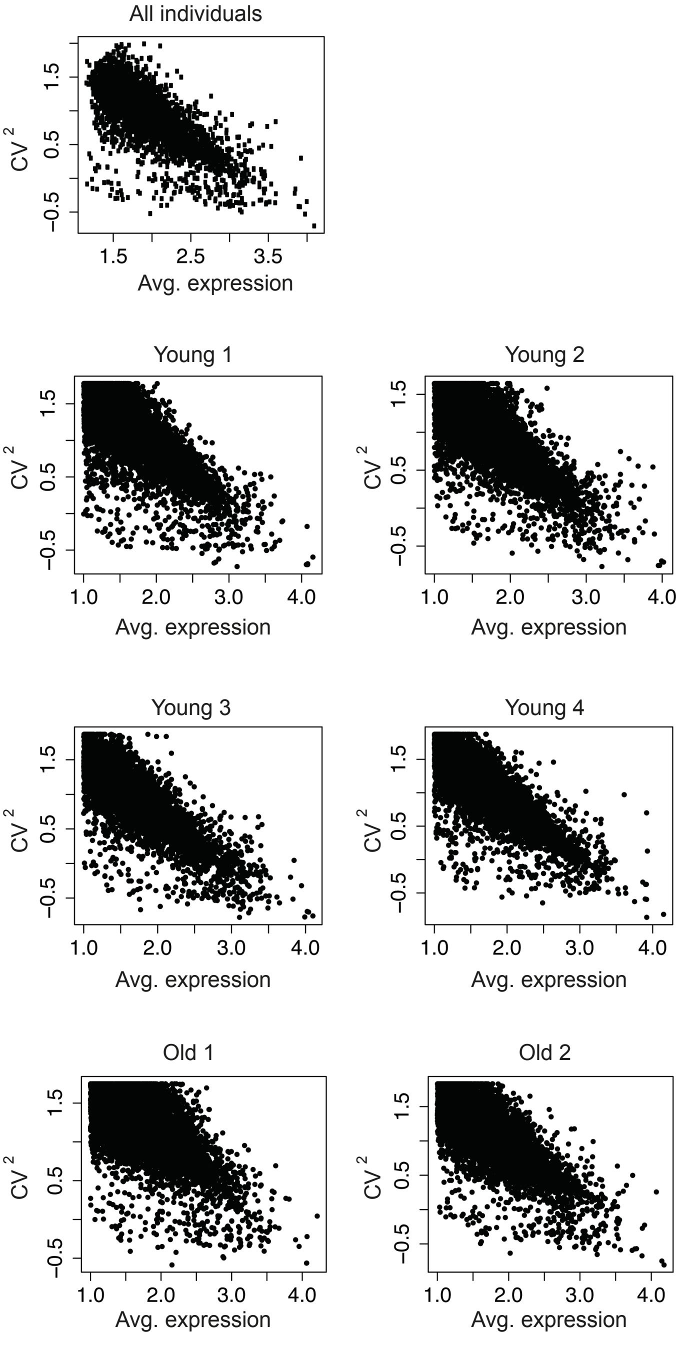

Supplementary Figure 19. Transcriptional variability among muscle stem cells from young and old mice.

Gene variability: squared coefficients of variation are plotted against the means of normalized read counts for gene using data from all individuals (top) or each individual separately.

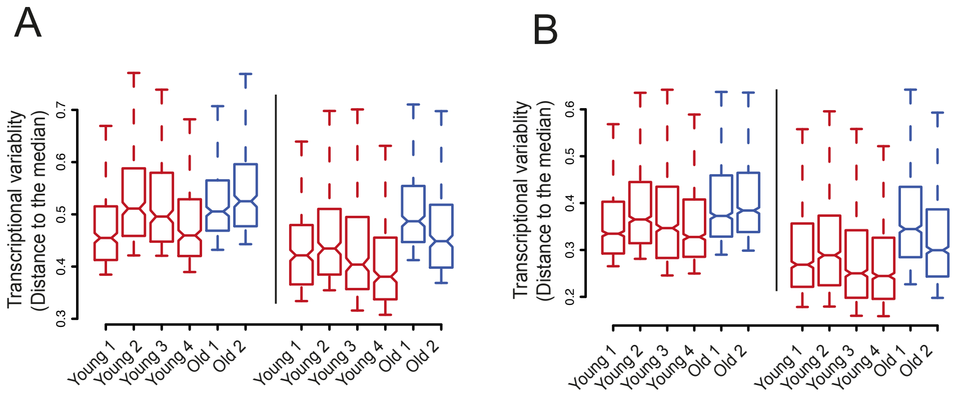

Supplementary Figure 20. Transcriptional variability: distance to the median

Distance to the median of the top 300 (A) and 1000 (B) most variable genes among all genes (left) and among the 5,127 common genes expressed in the six individuals (right). For all boxplots, the box represents the interquartile range and the horizontal line in the box represents the median (n=300).

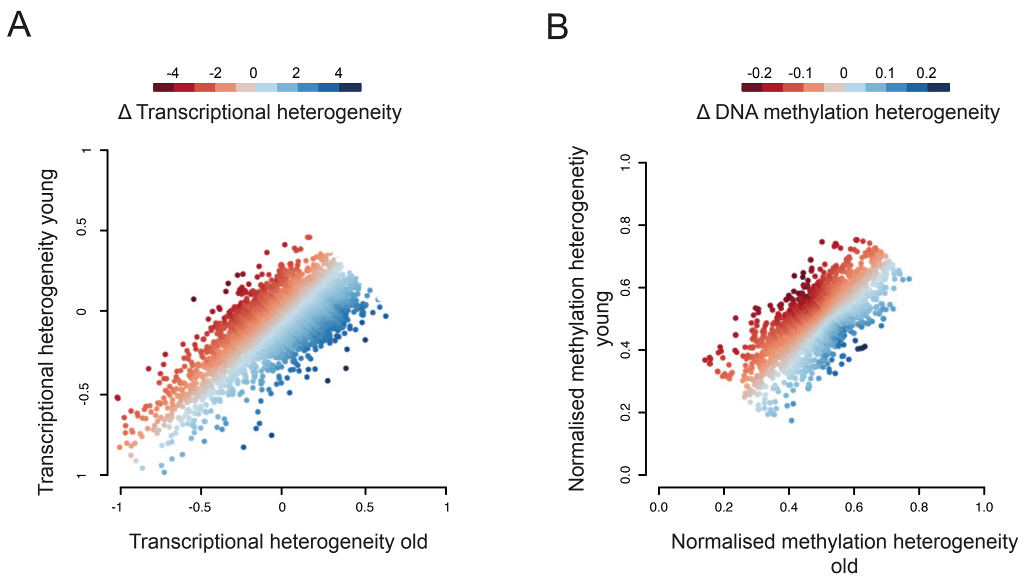

Supplementary Figure 21. Changes in transcriptional and DNA methylation heterogeneity with age.

(A) Differences in transcriptional heterogeneity measures where Z-score normalised using a sliding window of 100 observations (color code). Transcriptional heterogeneity represents the mean distance to the median for every gene from young (y-axis) and old (x-axis) individuals.

(B) Differences in DNA methylation heterogeneity measures where Z-score normalised using a sliding window of 100 observations (color code). DNA methylation heterogeneity represents the normalised measure of methylation heterogeneity from young (y-axis) and old (x-axis) individuals.

Supplementary Table 1. scM&T quality control

Pax7-nGFP Hi cells

						RNA		DNA	
Individual	Sequencing label	Sex	Genetic background	Age (weeks)	Age (months)	Total cells	After QC	Total cells	After QC
Young 1	Y8	Male	B6D2F1/JRj	6.9	1.6	96	75	86	35
Young 2	Y4	Male	B6D2F1/JRj	6.6	1.5	96	60	NA	NA
Young 3	Y5	Male	B6D2F1/JRj	6.6	1.5	96	44	NA	NA
Young 4	Y7	Male	B6D2F1/JRj	6.9	1.6	96	74	72	0
Young 5	Y2	Male	B6D2F1/JRj	6.6	1.5	96	0	78	35
Old 1	O1	Male	B6D2F1/JRj	114.1	26.3	96	56	80	35
Old 2	O5	Male	B6D2F1/JRj	114.1	26.3	96	68	90	35
Old 3	O8	Male	B6D2F1/JRj	117.3	27.0	96	5	8	0

Pax7-nGFP Total cells

						RNA	
Individual	Sequencing label	Sex	Genetic background	Age (weeks)	Age (months)	Total cells	After QC
Young 1	Y15	Male	B6D2F1/JRj	9.1	2.1	96	20
Young 2	Y21	Male	B6D2F1/JRj	9.1	2.1	96	35
Old 1	O16	Male	B6D2F1/JRj	101.4	23.3	96	56
Old 2	O17	Male	B6D2F1/JRj	101.4	23.3	96	36
