# Supplementary material for: Ageing affects DNA methylation drift and transcriptional cell-to-cell variability in mouse muscle stem cells
Source: Nat Commun. 2019 Sep 25;10:4361. doi: 10.1038/s41467-019-12293-4 (PMC6761124; doi:10.1038/s41467-019-12293-4)
Supplement: Supplementary file 3 — Description of Additional Supplementary Files [file 41467_2019_12293_MOESM3_ESM.pdf]

Description of Additional Supplementary Files

File Name: Supplementary Data 1
Description: Differentially expressed genes between cells from young and old mice.

File Name: Supplementary Data 2
Description: Top 1000 most variable genes across the entire data set.

File Name: Supplementary Data 3
Description: Top 200 genes correlated and anticorrelated with the similarity score to the young reference transcriptome.

File Name: Supplementary Data 4
Description: Increase of transcriptional and methylation heterogeneity with age in promoter regions (Δ : Old-young).
