# Supplementary material for: Ageing affects DNA methylation drift and transcriptional cell-to-cell variability in mouse muscle stem cells
Source: Nat Commun. 2019 Sep 25;10:4361. doi: 10.1038/s41467-019-12293-4 (PMC6761124; doi:10.1038/s41467-019-12293-4)
Supplement: Supplementary file 8 — Reporting Summary [file 41467_2019_12293_MOESM8_ESM.pdf]

nature research

Corresponding author(s): Shahragim Tajbakhsh, Wolf Reik, Irene Hernando-Herraez

# Reporting Summary

Nature Research wishes to improve the reproducibility of the work that we publish. This form provides structure for consistency and transparency in reporting. For further information on Nature Research policies, see Authors & Referees and the Editorial Policy Checklist.

## Statistical parameters

When statistical analyses are reported, confirm that the following items are present in the relevant location (e.g. figure legend, table legend, main text, or Methods section).

| n/a | Confirmed | |
|---|---|---|
| ☐ | ☒ | The exact sample size (*n*) for each experimental group/condition, given as a discrete number and unit of measurement |
| ☐ | ☒ | An indication of whether measurements were taken from distinct samples or whether the same sample was measured repeatedly |
| ☐ | ☒ | The statistical test(s) used AND whether they are one- or two-sided<br>*Only common tests should be described solely by name; describe more complex techniques in the Methods section.* |
| ☐ | ☒ | A description of all covariates tested |
| ☐ | ☒ | A description of any assumptions or corrections, such as tests of normality and adjustment for multiple comparisons |
| ☐ | ☒ | A full description of the statistics including central tendency (e.g. means) or other basic estimates (e.g. regression coefficient) AND variation (e.g. standard deviation) or associated estimates of uncertainty (e.g. confidence intervals) |
| ☐ | ☒ | For null hypothesis testing, the test statistic (e.g. *F*, *t*, *r*) with confidence intervals, effect sizes, degrees of freedom and *P* value noted<br>*Give P values as exact values whenever suitable.* |
| ☒ | ☐ | For Bayesian analysis, information on the choice of priors and Markov chain Monte Carlo settings |
| ☐ | ☒ | For hierarchical and complex designs, identification of the appropriate level for tests and full reporting of outcomes |
| ☐ | ☒ | Estimates of effect sizes (e.g. Cohen's *d*, Pearson's *r*), indicating how they were calculated |
| ☐ | ☒ | Clearly defined error bars<br>*State explicitly what error bars represent (e.g. SD, SE, CI)* |

*Our web collection on statistics for biologists may be useful.*

## Software and code

Policy information about availability of computer code

| | |
|---|---|
| Data collection | *Provide a description of all commercial, open source and custom code used to collect the data in this study, specifying the version used OR state that no software was used.* |
| Data analysis | Custom software is available upon request. |

For manuscripts utilizing custom algorithms or software that are central to the research but not yet described in published literature, software must be made available to editors/reviewers upon request. We strongly encourage code deposition in a community repository (e.g. GitHub). See the Nature Research guidelines for submitting code & software for further information.

## Data

Policy information about availability of data

All manuscripts must include a data availability statement. This statement should provide the following information, where applicable:

- Accession codes, unique identifiers, or web links for publicly available datasets
- A list of figures that have associated raw data
- A description of any restrictions on data availability

Raw and processed data is available in GEO under the accession number: GSE121364 . All other data are available from the authors upon request.



April 2018

# Field-specific reporting

Please select the best fit for your research. If you are not sure, read the appropriate sections before making your selection.

☒ Life sciences ☐ Behavioural & social sciences ☐ Ecological, evolutionary & environmental sciences

For a reference copy of the document with all sections, see nature.com/authors/policies/ReportingSummary-flat.pdf

# Life sciences study design

All studies must disclose on these points even when the disclosure is negative.

| | |
|---|---|
| Sample size | No statistical methods were used to predetermine sample size. Sample size was determined by limited availability of cells. |
| Data exclusions | No data was excluded from the study. |
| Replication | We use at least 2 young and 2 old mice. |
| Randomization | As a general rule, mice (young or old) were littermates. |
| Blinding | Investigators were not blinded. |

# Reporting for specific materials, systems and methods

## Materials & experimental systems

| n/a | Involved in the study | |
|---|---|---|
| ☒ | ☐ | Unique biological materials |
| ☐ | ☒ | Antibodies |
| ☒ | ☐ | Eukaryotic cell lines |
| ☒ | ☐ | Palaeontology |
| ☐ | ☒ | Animals and other organisms |
| ☒ | ☐ | Human research participants |

## Methods

| n/a | Involved in the study | |
|---|---|---|
| ☒ | ☐ | ChIP-seq |
| ☐ | ☒ | Flow cytometry |
| ☒ | ☐ | MRI-based neuroimaging |

## Antibodies

| | |
|---|---|
| Antibodies used | Anti-BrdU antibody, Becton Dickinson, #347580<br>Anti-M-Cadherin antibody, Nanotools, clone 12G4<br>Anti-ItgB1 antibody, SantaCruz, #sc-9936<br>Anti-GFP antibody, Abcam, #ab13970<br>Anti-chicken AlexaFluor 488 antibody, ThermoFisher, #A-11039<br>Anti-mouse Cy3 antibody, Jackson ImmunoResearch, #115-165-205<br>Anti-goat DyLight 550 antibody, Diagomics, #DKXGT-003 |
| Validation | Anti-BrdU antibody: validated in vitro on proliferating cells treated or not with BrdU.<br><br>Anti-M-Cadherin antibody: expected staining pattern as notably reported by Goel et al., Cell Reports, 2017<br><br>Anti-ItgB1 antibody: expected staining pattern as notably reported by Rozo et al., Nature Medicine, 2016<br><br>Anti-GFP antibody: validated on isolated single muscle fibers from WT and Tg:Pax7-nGFP mice. |

## Animals and other organisms

Policy information about studies involving animals; ARRIVE guidelines recommended for reporting animal research

| | |
|---|---|
| Laboratory animals | Mus musculus Tg:Pax7-nGFP (MGI:5308730), males, 2 or 24 months-old |
| Wild animals | The study did not involve wild animals |

Field-collected samples | The study did not involve samples collected from the field

## Flow Cytometry

### Plots

Confirm that:

☒ The axis labels state the marker and fluorochrome used (e.g. CD4-FITC).

☒ The axis scales are clearly visible. Include numbers along axes only for bottom left plot of group (a 'group' is an analysis of identical markers).

☒ All plots are contour plots with outliers or pseudocolor plots.

☒ A numerical value for number of cells or percentage (with statistics) is provided.

### Methodology

Sample preparation: Tibialis anterior muscles were dissected and placed into cold DMEM. Muscles were then chopped and put into a 15 ml Falcon tube containing 10 ml of DMEM, 0.08% collagenase D, 0.1% trypsin, 10 µg/ml DNaseI at 37°C under gentle agitation for 25 min. Digests were allowed to stand for 5 min at room temperature and the supernatants were collected on 5 ml of foetal bovine serum (FBS) on ice. The digestion was repeated 3 times until complete digestion of the muscle. The supernatants were filtered through a 70-µm cell strainer. Cells were spun for 15 min at 515g at 4°C and the pellets were resuspended in 1 ml freezing medium (10% DMSO in foetal calf serum (FCS) for long term storage in liquid nitrogen. Before isolation by FACS, samples were thawed in 50 ml of cold DMEM, spun for 15 min at 515g at 4°C. Pellets were resuspended in 300 µl of DMEM 2% FCS 1 µg/mL propidium iodide and filtered through a 40-µm cell strainer.

Instrument: MoFlo Astrios cell sorter (Beckmann Coulter)

Software: Summit v6.3.1

Cell population abundance: Single cells were sorted with the following parameters: Abort mode Single / drop enveloppe 0.5

Gating strategy: Satellite cells were identified gating first on known size and granulosity (FSC-Height vs SSC-Height), excluding doublets (FSC-Height vs FSC-Area), and gating on viable (propidium iodide-negative) GFP-positive population (561-579/16-Height vs 488-513/26-Height). Pax7-nGFP High cells (top 10% highest nGFP-expressing cells) were sorted as single cells.

Propidium iodide (PI)-positivity was set based on a non-PI-treated aliquot of the same sample.
nGFP-positivity was set based on a WT (ie non Tg:Pax7-nGFP) muscle sample.

☒ Tick this box to confirm that a figure exemplifying the gating strategy is provided in the Supplementary Information.
